# Targeting the Histone Methyltransferase Disruptor of Telomeric Silencing 1-Like Restricts Avian Leukosis Virus Subgroup J Replication by Restoring the Innate Immune Response in Chicken Macrophages

**DOI:** 10.3389/fmicb.2020.603131

**Published:** 2020-12-08

**Authors:** Shihao Chen, Dedong Wang, Yinyin Liu, Ruihan Zhao, Ting Wu, Xuming Hu, Zhiming Pan, Hengmi Cui

**Affiliations:** ^1^Institute of Epigenetics and Epigenomics and College of Animal Science and Technology, Yangzhou University, Yangzhou, China; ^2^Jiangsu Key Laboratory of Zoonosis, Yangzhou University, Yangzhou, China; ^3^Joint International Research Laboratory of Agricultural and Agri-Product Safety, The Ministry of Education of China, Yangzhou University, Yangzhou, China; ^4^College of Veterinary Medicine, Yangzhou University, Yangzhou, China; ^5^Poultry Institute, Chinese Academy of Agricultural Sciences, Yangzhou, China; ^6^Institute of Comparative Medicine, Yangzhou University, Yangzhou, China

**Keywords:** DOT1L, ALV-J, innate immunity, MDA5, chicken macrophages

## Abstract

Avian leukosis virus subgroup J (ALV-J), an oncogenic retrovirus, is known to cause immunosuppression and various types of cancer in chickens. Recent reports have shown that epigenetic changes in DNA and chromatin are widely implicated in the life cycle of diverse viruses, and reversal of these changes in host cells can lead to alterations in the propagation of viruses. In the present study, we found that disruptor of telomeric silencing 1-like (DOT1L), a histone H3 lysine79 (H3K79) methyltransferase, was upregulated during ALV-J infection in chicken macrophage HD11 cells. Subsequently, we show that targeting DOT1L with a specific inhibitor can significantly decrease the ALV-J replication and viral production. By generating of DOT1L-knockout (KO) HD11 cells using the CRISPR/Cas9 system, we show that deletion of the DOT1L led to an increase in the induction of *IFNβ* and interferon-stimulated genes (ISGs) in HD11 cells in response to ALV-J infection. Importantly, we confirmed that ALV-J infection impaired the activation of the melanoma differentiation-associated protein 5 (MDA5)-mediated-IFN pathway by suppressing the MDA5 expression, and knockout DOT1L rescued the expression of MDA5 and signal transducer and activator of transcription 1 (STAT1), both of which tightly control the antiviral innate immunity. Collectively, it can be deduced from the current data that blocking DOT1L activity or deletion of DOT1L can lead to ALV-J replication inhibition and restoration of the virally suppressed host innate immunity. Thus, we suggest that DOT1L might be a potential drug target for modulating host innate immune responses to combat ALV-J infection.

## Introduction

Avian leukosis virus subgroup J (ALV-J) is an enveloped RNA virus belonging to the alpha-retrovirus genus of the retrovirus family (Payne and Nair, 2012). ALV-J spreads widely in both commercial‐ and meat-type chickens throughout the world. In the past decade, it was found that ALV-J has also spread to egg-type chickens (Liu et al., 2011). ALV-J infection not only causes lymphoid and myeloid leukosis and several other types of tumors, but also leads to long-term immunosuppression, which is a major contributing factor to morbidity and mortality in some chicken flocks (Gao et al., 2015). ALV-J infection has caused enormous economic losses to the poultry industry worldwide in recent years (Gao et al., 2010; Payne and Nair, 2012). Currently, there is still no effective vaccine or specific antiviral drug to combat ALV-J infection.

Epigenetic modifications, such as DNA methylation and histone modification, have been reported to contribute to fundamental biological processes, and epigenetic dysregulation has been liked to several human diseases such as cancer. Thus, epigenetic inhibitors that reverse aberrant epigenetic modifications in cancer cells have been developed for clinical treatment. For instance, histone deacetylase (HDAC) inhibitors, such as vorinostat and panobinostat, have been approved by the United States Food and Drug Administration (FDA) for cancer treatment (Baskin and Haynes, 2019). More recently, several reports have shown that epigenetic modifications can also modulate immunological and inflammatory processes, including innate immunity and antiviral responses (Li et al., 2016; Xue et al., 2016; Chen et al., 2018; Zhang et al., 2018). As a consequence, targeting epigenetic regulators as a novel antiviral strategy is being explored (Boehm and Ott, 2017; Marcos-Villar et al., 2018). In our previous study, we found that the DNA methyltransferase (DNMT) inhibitor 5-aza-2'-deoxycytidine shows antiviral activity against ALV-J infection by inducing high expression of an antisense lncRNA that can activate the host defense (Chen et al., 2019). The ALV-J genome contains two copies of single-stranded RNA with positive polarity. Similar to other retroviruses, upon ALV-J infection, the single-stranded RNA viral genome is converted into a double-stranded DNA that subsequently integrates into the host genome (Lee et al., 2019). Therefore, there is no doubt that ALV-J infection can alter the host chromatin state such as chromatin accessibility, which probably facilitates its infection. However, little is known about the cellular chromatin regulators involved in ALV-J infection.

Disruptor of telomeric silencing 1-like (DOT1L) is a unique histone methyltransferase that catalyzes the mono-, di-, and tri-methylation of lysine-79 of histone H3 (H3K79me1/2/3). DOT1L exhibits a variety of chromatin-associated functions in mammalian cells, including transcriptional regulation (Cattaneo et al., 2016), cell cycle regulation (Kim et al., 2014), heterochromatin formation (Sarno et al., 2020), and DNA repair (Nguyen and Zhang, 2011). Moreover, DOT1L was found to be significantly associated with the initiation and progression of the mixed-lineage leukemia (MLL)-rearranged leukemia and other solid tumors (Cho et al., 2015; Sarno et al., 2020). Based on these actions, some compounds, such as EPZ004777 and EPZ5676, that target the catalytic activity of DOT1L have been developed for the treatment of MLL-induced leukemia as well as other solid tumors (Stein et al., 2018; Yang et al., 2019; Sarno et al., 2020).

In the present study, we observed that the mRNA and protein expression of DOT1L were significantly upregulated in ALV-J infected HD11 cells. An inhibitor of DOT1L, EPZ004777, can significantly decrease the ALV-J replication and viral production. Subsequently, by constructing DOT1L-knockout (KO) HD11 cells using CRISPR/Cas9, we showed that deletion of the DOT1L led to an increase in the induction of the expression of *IFNβ* and interferon-stimulated genes (ISGs) in HD11 cells in response to ALV-J infection. Consequently, viral yields in DOT1L-KO cells were significantly diminished with increasing the expression of these antiviral ISGs. Collectively, it can be deduced from the current data that blocking DOT1L activity or deletion of DOT1L can lead to ALV-J replication inhibition and restoration of virally suppressed host innate immunity. DOT1L might serve as a potential drug target by modulating host innate immune responses to combat ALV-J infection.

## Materials and Methods

### Cells and Virus

The chicken macrophage HD11 cell line (kindly provided by Prof. Zhiming Pan, Yangzhou University, Yangzhou, China) and the chicken embryonic fibroblast DF-1 cell line (from American Type Culture Collection, Cat# CRL-12203) were grown in Dulbecco’s modified Eagle’s medium (DMEM; Thermo Scientific, Rockford) supplemented with 10% fetal bovine serum (FBS; Gibco, Paisley), 100 U/ml penicillin and 100 μg/ml streptomycin. Cells were incubated in a humidified 5% CO_2_ incubator at 37°C. The ALV-J strain JS09GY3 (accession number: GU982308.1) was kindly provided by Prof. Aijian Qin (Yangzhou University, China) and was propagated in DF-1 cells.

### Determination of Viral Titers

HD11 cells or DOT1L-KO cells (1 × 10^5^ cells/ml) were seeded on 12-well plates and cultured for 24 h before infection with ALV-J GY3 strain at the indicated titers. The culture supernatants were collected at the indicated time points after infection. The viral titers were determined by 50% tissue culture infective doses (TCID_50_) on DF-1 cells. ALV-J was not detected in the control HD11 or DOT1L-KO cells.

### Plasmids Construction

Recombinant Cas9-T2A-GFP plasmid used in this study was generated by inserting a T2A-GFP cassette to the C-terminus of SpCas9 (MLM3613, Addgene plasmid #42251). Three guide RNAs specific for the chicken DOT1L gene (GeneID: 84444) were designed using CHOPCHOP web tool,^1^ were then respectively cloned into U6-sgRNA plasmid (Addgene plasmid #65626). The oligo sequences for guide RNAs targeting DOT1L are listed in Table 1.

**Table 1:** Oligonucleotides used for plasmid construction.

Name		5'-3' sequence
chDOT1L-gRNA1	Forward	caccGTTGTTGAGTTTCTCGGGGT
chDOT1L-gRNA2	Forward	caccgCTCTTTGTCGACTTGGGCAG
chDOT1L-gRNA3	Forward	caccGAGGTTTCTCCATACACCTC

### CRISPR/Cas9-Mediated DOT1L Gene Knockout in HD11 Cells

The clonal DOT1L-KO HD11 cell lines were generated as following. Briefly, DF-1 cells seeded in six wells were co-transfected with Cas9-T2A-GFP plasmid and four guide RNA plasmids, respectively. Cells were incubated for 48 h, and the T7E1 assay was performed to select the sgRNAs with higher cleavage efficiency. Then, 9 μl of transfection reagent was used to transduce HD11 cells seeded in a 6-cm dish with 3 μg of Cas9-T2A-GFP plasmid and selected sgRNA. Cells were incubated for 48 h, and then GFP-expressing cells were sorted into single wells of a 96-well dish containing DMEM with 15% FBS on a BD InFlux cell sorter. Plates were cultured for 3 weeks for colony outgrowth. These clones were further identified for homozygous frame-shift mutation using genomic DNA PCR. Lysates were prepared and immunoblotted as described below to identify knockout cell lines.

### RNA-Seq Analysis

HD11 cells or DOT1L-KO cells grown into a monolayer were infected with ALV-J (MOI 2). Twelve hours later, the cells were harvested, and total RNA was isolated from the HD11 and DOT1L-KO cells infected with ALV-J. The concentration and integrity of the RNA samples were confirmed using a 2100 Bioanalyzer (Agilent Technologies). A total amount of 1 μg RNA per sample was used as input material for the RNA-seq library preparations, which were generated using NEBNext Ultra™ RNA Library Prep Kit for Illumina (NEB, United States) following the manufacturer’s recommendations. The RNA sequencing (RNA-seq) was performed on Illumina HiSeq2000 platform at BIOMARKEER Biotech Comp (Beijing, China). High-quality reads were aligned to the chicken genome (gga5.0) using TopHat v2.0.9. The expression levels for each of the genes were normalized to fragments per kilobase of transcript per million (FPKM fragments mapped). Cuffdiff was used to compare mRNA levels between samples. The FDR < 0.05 and fold change ≥ 1.5 were set as the threshold for significantly differential expression. We used KOBAS software to test the statistical enrichment of the differential expression genes in KEGG pathways. The MA plot and heatmap for the study were generated by the open-source R programming.

### Quantitative Real-Time Reverse Transcription PCR

The mRNA levels for genes examined, including *DOT1L*, *ENV*, *IFIH1*, *IFNβ*, *STAT1*, and *GAPDH* genes, in HD11 and DOT1L-KO cells with indicated infection or treatment were analyzed by a two-step real-time RT-PCR. Total RNA was extracted from the cells with TRIzol reagent (Takara, Japan) following the manufacturer’s instructions. One microgram of the total RNA was used for cDNA synthesis using a Vazyme cDNA Synthesis Kit (Vazyme, Nanjing, China). The BioRad CFX Connect Real-time PCR detection system (BioRad) was used to perform qPCR with ChamQ SYBR qPCR Master Mix Kit (Vazyme). PCR reactions were performed in a total volume of 20 μl containing 2 μl of diluted cDNA, 10 μl of ChamQ SYBR qPCR Master Mix, and4 μl of primers mix (1 μM of each gene specific primer). The PCR conditions used were 95°C for 30 s, followed by 40 cycles of 10 s at 95°C and 30 s at 60 °C, as recommended by the manufacturer. The relative expression levels for the tested mRNAs were calculated using the 2^−∆∆Ct^ method and normalized to the *GAPDH* gene. qPCR primer sequences are provided in Table 2.

**Table 2:** Primers used in qRT-PCR.

Gene		5'-3' sequence
*GAPDH*	Forward	GAGAAACCAGCCAAGTATGA
	Reverse	CTGGTCCTCTGTGTATCCTA
*DOT1L*	Forward	GGAGCAGGAGAAGGAGAA
	Reverse	AATCAGGTCGTTGTATGTCA
ALV-J *Env*	Forward	TGCGTGCGTGGTTATTATTTC
	Reverse	AATGGTGAGGTCGCTGACTGT
*IFIH1*	Forward	GTGTCCGCTTGTCAGATT
	Reverse	AGGTGAGGCTGTAAGTCC
*IFNβ*	Forward	GCCCACACACTCCAAAACACTG
	Reverse	TTGATGCTGAGGTGAGCGTTG
*STAT1*	Forward	CTTGATGCTGGGAGAGGAGT
	Reverse	TGAGGGAGAGAGAGCGAAAG
*MX1*	Forward	CCGCAACACAGAAATACAG
	Reverse	TTATCTTGTGGCTGGTTCC

### Salt-Soluble Protein Extraction and Histone Extraction

For salt-soluble protein extraction, cells were lysed for 15 min on ice with cell lysis buffer (Cell Signaling Technology) supplemented with protease inhibitors. The cell suspension was sonicated two times using 5 s on/5 s off at 25% power on a sonicator (SONICS). The supernatant were then processed for western blot analysis. For histone extraction, histone proteins were extracted from nuclear pellets according to the instruction followed by EpiQuik Total Histone Extraction Kit (Cat#OP-0006-100, EPIGENTEK).

### Western Blotting

The proteins were separated by SDS-PAGE, transferred to a nitrocellulose membrane, and then blocked for 1 h at room temperature (RT). The membranes were incubated overnight at 4°C with the appropriate primary antibodies: mouse monoclonal antibody JE9 (kindly provided by Prof. Aijian Qin, Yangzhou University, China), DOT1L (cat#A11285, ABclonal), histone H3 lysine 79 di-methylation (H3K79me2; cat#ab3594, Abcam), histone H3 (cat#A2348, ABclonal), TANK binding kinase 1 (TBK1; cat#04-856, Millipore), phosphorylated TBK1 (cat#5483, Cell Signaling Technology), STAT1 (cat#AV38933, Sigma), MDA5 (cat#A13645, ABclonal), *β*-actin (cat#ab8226, Abcam), and *α*-tubulin (cat#PM054, MBL) followed by HRP-linked secondary anti-rabbit (cat#458, MBL) or anti-mouse secondary antibodies (cat#330, MBL). The proteins were visualized by NcmECL ultra substrate (cat#P10200B, NCM) in accordance to the manufacturer-specified protocol. The images were collected with a Western-blot imaging system (FlourChemQ, ProteinSimple). For quantification of the data, the bands were analyzed using Image J software.

### Indirect Immunofluorescence Assay

Cells were fixed in ice-cold acetone: ethanol (3:2, v/v) mixture for 10 min and were washed with PBS. The cells were then incubated at room temperature with the JE9 mAb (anti-Env) for 2 h, followed by incubation with Alexa Fluor 488-conjugated AffinPure Goat Anti-Mouse IgG (H + L; cat#AS076, ABclonal) for 45 min and stained with DAPI (cat#D-9542, Sigma-Aldrich) at room temperature for an additional 2 min. After three washes with PBS, images were captured and merged under an inverted fluorescence microscope.

### Statistical Analysis

The statistical analysis was performed with GraphPad Prism version 8.0 (GraphPad software). Data are represented as mean ± SD from three independent experiments. Statistical significance was assessed by the Student’s *t*-test for two groups. Differences with *p* < 0.05 were considered statistically significant.

## Results

### DOT1L Is Upregulated During ALV-J Infection in Chicken HD11 Cells

**Figure 1:**
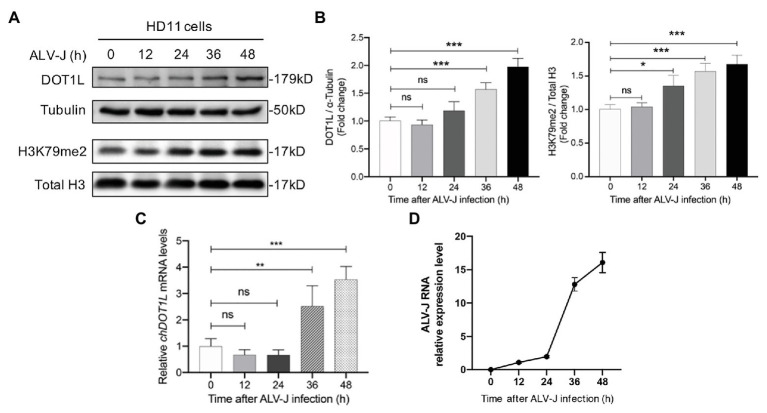
Disruptor of telomeric silencing 1-like (DOT1L) expression and H3K79me2 levels are upregulated during avian leukosis virus subgroup J (ALV-J) infection in chicken macrophage cell line HD11. **(A,B)** Western blot **(A)** and intensity **(B)** for DOT1L protein and DOT1L-mediated H3K79me2 levels in HD11 cells at the indicated time points post infection with ALV-J (MOI 2). *α*-tubulin and total H3 were used as a loading control. **(C)** The relative *DOT1L* mRNA levels in HD11 cells at the indicated time points post infection with ALV-J, measured by quantitative real-time reverse transcription PCR (qRT-PCR). GAPDH was used as a normalizing control. **(D)** The relative ALV-J RNA level in HD11 cells at the indicated time points post infection, normalized to chicken GAPDH mRNA, was measured by qRT-PCR. All the data were shown as mean ± SD (error bars) from three independent experiments. ns, not significant; ^*^p < 0.05; ^**^p < 0.01; ^***^p < 0.001; unpaired, two-tailed Student’s *t*-test.

We firstly analyzed the expression of DOT1L in HD11 cells in response to the infection with ALV-J. Western blotting analysis was performed to measure the expression levels of DOT1L in mock and ALV-J-infected HD11 cells over a 48 h time course (Figures 1A,D). The results suggested that the expression of DOT1L was significantly increased at 36 and 48 hpi compared with that in the mock-infected cells (Figure 1A). Accordingly, the level of DOT1L-mediated H3K79me2 also significantly increased at 24, 36, and 48 hpi (Figure 1B). Consistent with the results for the DOT1L protein, the relative mRNA levels of DOT1L also showed a significant increase at 36 and 48 hpi compared to those in cells with mock infection (Figure 1C).

### Chemical Blockade of DOT1L Promotes ALV-J Clearance in HD11 Cells

**Figure 2:**
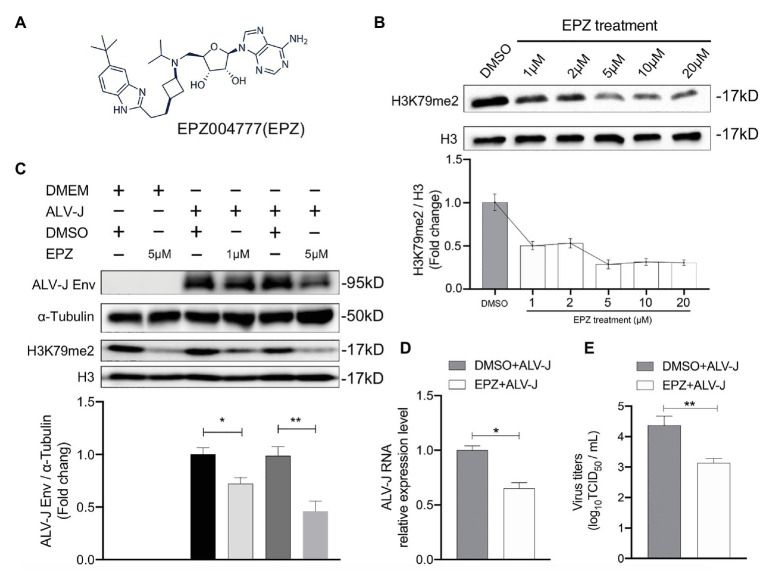
Chemical blockade of DOT1L promotes ALV-J clearance in HD11 cells. **(A)** Chemical structure of DOT1L inhibitor EPZ004777 (EPZ). **(B)** The blocking efficiency of EPZ was evaluated by western blotting. HD11 cells were incubated with various concentrations of EPZ or the vehicle control DMSO for 48 h, and the levels of H3K79me2 was determined by western blot (upper panel). Total histone H3 were used as internal control. Relative protein levels were quantified with ImageJ software and normalized to the amount of total H3 (lower panel). **(C)** Western blot analysis of ALV-J Env protein. HD11 cells were infected with ALV-J (MOI 2) in the presence of indicated concentrations of EPZ or vehicle control. ALV-J Env protein and the levels of H3K79me2 was determined at 48 hpi. *β*-actin and histone H3 were used as internal control (upper panel). Relative protein levels of ALV-J Env were quantified with ImageJ software and normalized to the amount of β-Actin (lower panel). HD11 cells were incubated with 5 μM of EPZ004777 for 24 h and then infected with ALV-J (MOI 2) for 48 h. Viral RNA levels were measured by qRT-PCR. GAPDH mRNA level was measured as an internal control **(D)**.Viral RNA was Viral yield in the supernatants was analyzed by (TCID_50_) assay at 48 h after infection **(E)**. All the data were shown as mean ± SD (error bars) from three independent experiments. ^*^p < 0.05; ^**^p < 0.01; unpaired, two-tailed Student’s *t*-test.

We next investigated whether DOT1L affected host cell susceptibility to ALV-J viruses. The DOT1L-specific inhibitor, EPZ004777, was used to block the DOT1l activity (Figure 2A). First, we treated HD11 cells with concentrations of EPZ004777 ranging from 1 to 20 μM for 48 h, and then detected the expression of H3K79me2, which was catalyzed by DOT1L, to confirm that there was a suitable drug concentration in chicken HD11 cells (Figure 2B). Cell-Counting Kit-8 (CCK-8) detection suggests that the treatment with 20 μM of EPZ004777 did not significantly change the cell viability of HD11 compared with that in the vehicle control dimethyl sulfoxide (DMSO) group (data not shown). The western blotting results show that the level of H3K79me2 decreased in a dose-dependent manner when the concentrations of EPZ004777 was lower than 5 μM, whereas the level of H3K79me2 showed no further reduction at higher concentrations (10 or 20 μM) of EPZ004777 (Figure 2B). Therefore, 5 μM was selected as the maximum concentration used for EPZ004777 treatment in this study.

We found that inhibiting DOT1L in ALV-J-infected HD11 cells, with the specific inhibitor EPZ004777 at 1 or 5 μM, significantly suppressed the production of the ALV-J envelope (Env) protein required for virus replication (Figure 2C). To further characterize the antiviral effect of Dot1l in host cells, we determined the RNA levels and virus titers of ALV-J through qRT-PCR and TCID_50_ determination, respectively. Our results show that the treatment with 5 μM of EPZ004777 significantly decreased ALV-J RNA levels and viral titers in the infected HD11 cells compared with those in the vehicle control DMSO cells (Figures 2D,E). Overall, EPZ004777 appears to reduce the levels of viral RNA and proteins during the ALV-J lifecycle, suggesting that chemically blocking DOT1L activity can significantly suppress ALV-J replication.

### Generation of DOT1L-KO HD11 Cells Using the CRISPR/Cas9 System

**Figure 3:**
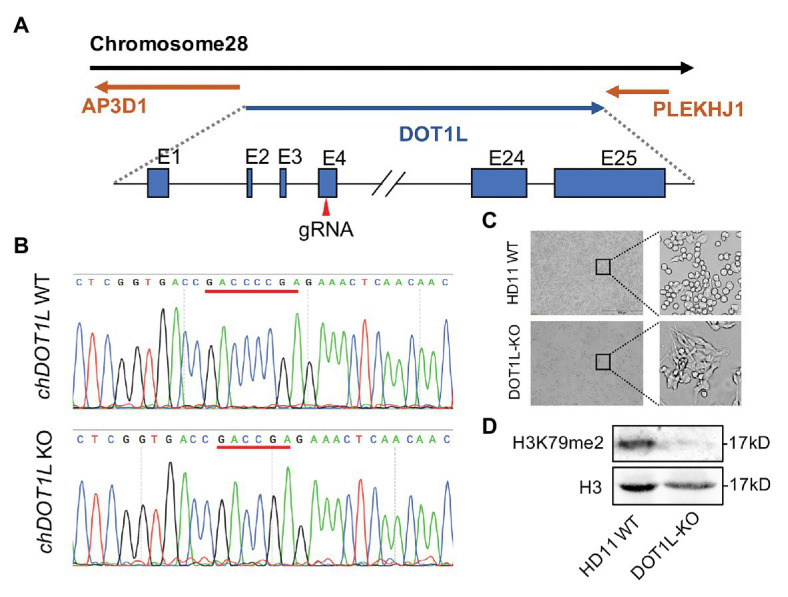
Generation and identification of DOT1L-knockout HD11 cell line. **(A)** The genomic architecture of chicken DOT1L gene and the CRISPR/cas9 strategy for DOT1L knockout. **(B)** DNA sequencing results of the chicken DOT1L allele between the wild-type (WT) and the DOT1L knockout cells clone **(C)** HD11 morphology after DOT1L depletion. Scale bar, 500 μm. **(D)** Western blot detected the levels of DOT1L mediated-H3K79me2 in DOT1L-KO cells and WT cells.

To explore the role of DOT1L in response to ALV-J infections, we knocked out DOT1L in HD11 cells with the CRISPR/Cas9 technique (Figure 3A). The sequencing results showed that sgRNA targeting of cells generated a reading frame shift mutants with a 2 bp deletion in exon 4 of the *DOT1L* gene (Figures 3A,B). Interestingly, morphological differences between wild-type (WT) and DOT1L knockout HD11 cells were observed (Figure 3C), which could be explained by the fact that DOT1L expression is involved in controlling cell differentiation and plasticity (Breindel et al., 2017). Subsequently, immunoblot analysis further confirmed the complete loss of DOT1L-mediated H3K79 methylation in the WT and homozygous mutant (DOT1L-KO) macrophages (Figure 3D).

### Knockout of DOT1L Limits ALV-J Replication

**Figure 4:**
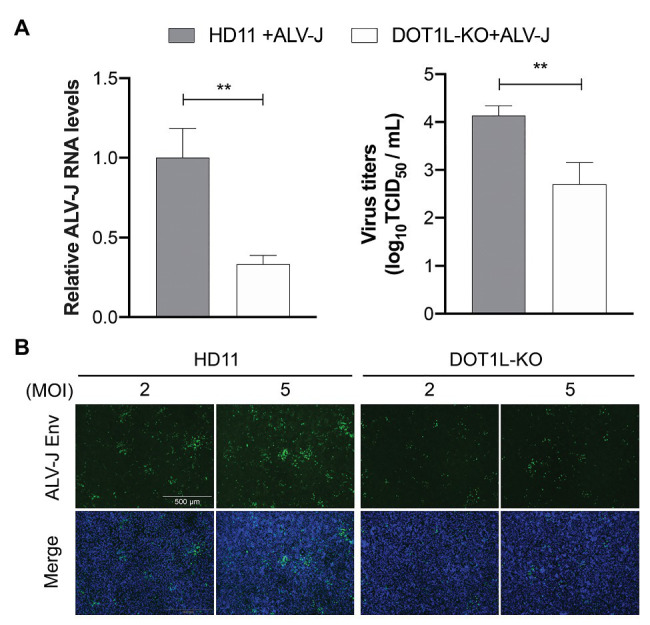
Knockout of DOT1L inhibits ALV-J replication. **(A)** HD11 and DOT1L-KO cells were infected with ALV-J (MOI 2). After 48 h, the viral RNA levels were measured by qRT-PCR. GAPDH mRNA level was measured as an internal control (left panel). The supernatants were collected to measure the viral titers by a standard TCID_50_ method (right panel). **(B)** Immunofluorescence analyses of ALV-J Env protein. HD11 cells and DOT1L-KO cells were infected with ALV-J at indicated MOI for 48 h, the cells were then visualized with the inverted fluorescence microscope and a specific antibody to the ALV-J Env protein (green), and nuclei were stained with 4', 6-diamidino-2-phenylindole (DAPI; blue). Images show a representative image. All the data were shown as mean ± SD (error bars) from three independent experiments. ^**^p < 0.01; unpaired, two-tailed Student’s *t*-test.

To examine whether DOT1L knockout caused chicken macrophages to clear ALV-J viruses, as observed in the cells treated with the DOT1L inhibitor, we analyzed the RNA expression and viral titers of ALV-J in infected HD11 cells. We observed a decreased amount of ALV-J RNA by qRT-PCR and decreased viral titers by TCID_50_ determination in DOT1L-KO HD11 cells compared to those in WT cells (Figure 4A). Consistently, the indirect immunofluorescence assay (IFA) confirmed a reduction in ALV-J Env fluorescence in DOT1L-KO cells compared to that in WT cells (Figure 4B).

### Gene Expression Analysis of HD11 and DOT1L-KO Cells Upon ALV-J Stimulation

**Figure 5:**
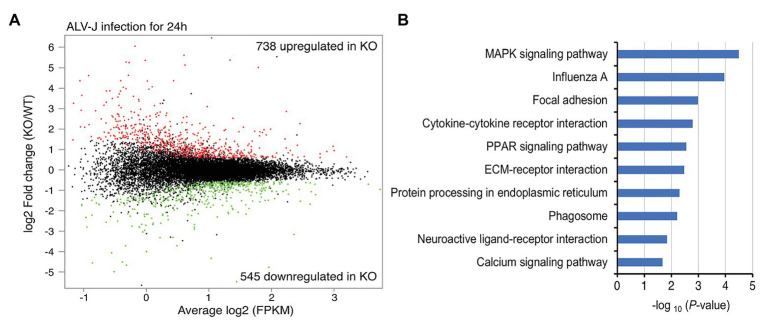
Gene-expression profiling of HD11 cells and DOT1L-KO cells upon ALV-J stimulation. **(A)** Mean average (MA) plot of genes differentially expressed in DOT1L-KO cells relative to their expression in HD11 cells. The log2 FPKM and log2 fold change between DOT1L-KO cells and WT cells are represented in the *x* and *y* axes, respectively. The red and green dots, respectively, indicate the upregulated and downregulated genes, and the black dots indicate no change in expression. FPKM, fragments per kilobase of transcript per million mapped reads. **(B)** KEGG pathway analysis showing the top 10 enriched pathways.

To elucidate the mechanism connecting DOT1L inhibition to enhanced clearance of ALV-J, we investigated the mRNA expression profiles of HD11 and DOT1L-KO cells infected with ALV-J for 24 h. Transcriptomic analysis revealed that DOT1L deletion by CRISPR/Cas9 system significantly altered the gene expression profile. A total of 738 genes were upregulated, and 545 genes were downregulated in DOT1L-KO cells compared with WT HD11 cells (Figure 5A). Unexpectedly, we found that the loss of DOT1L-H3K79 methylation, an activating marker, led to an increase in upregulated genes compared to downregulated genes in HD11 cells in response to ALV-J infection. Differential expression analysis showed that pro-inflammatory and viral infection-related response pathways, including the MAPK, influenza A, and cytokine-cytokine receptor interaction pathways, were ranked among the top 10 KEGG pathways (Figure 5B), indicating the presence of a defensive response to viral infection in DOT1L KO cells after ALV-J infection.

### Deficiency of DOT1L Elevated MDA5-Mediated Type I Interferon Signaling Following ALV-J Infection

**Figure 6:**
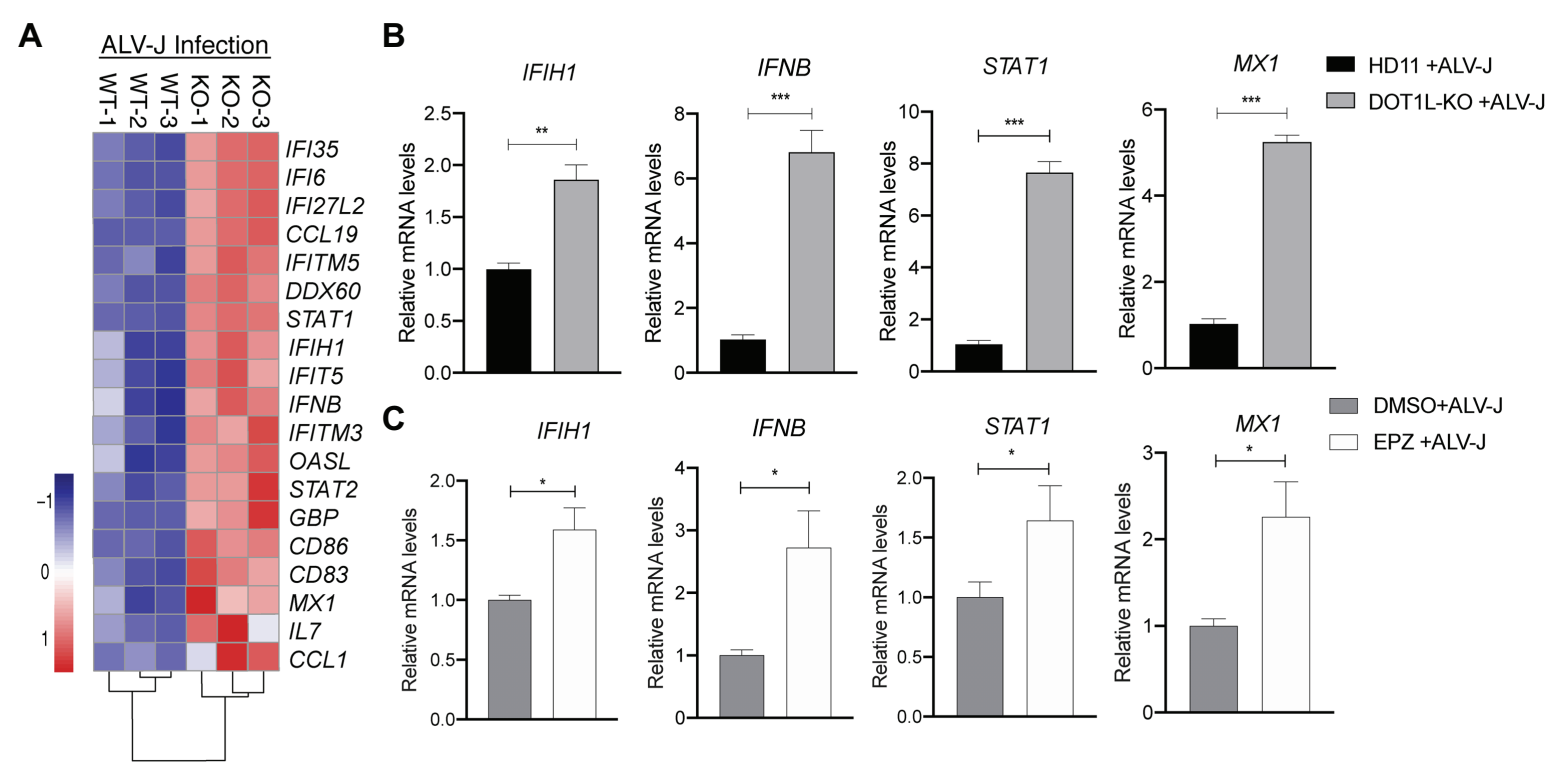
Knockout of DOT1L promotes defense response to ALV-J infection. **(A)** Heatmap of representative genes involved in type I IFN‐ related genes from DOT1L-KO vs. control cells infected with ALV-J at 24 h. **(B)** qRT-PCR validations. **(C)** HD11 cells were infected with ALV-J (MOI 2) in the presence of 5 μM EPZ or vehicle control (DMSO). At 48 hpi, the relative expression of *IFIH1*, *IFNβ*, *STAT1*, and *MX1* were determined by qRT-PCR. GAPDH were used as internal control. All the data were shown as mean ± SD (error bars) from three independent experiments. ^*^p < 0.05; ^**^p < 0.01; ^***^p < 0.001; unpaired, two-tailed Student’s *t*-test.

Importantly, in the RNA-seq analysis, we found many genes associated with the MDA5 (encoded by *IFIH1*)-mediated Type I interferon signaling pathway were upregulated in DOT1L-KO cells following ALV-J infection (Figure 6A). Obviously, it will be beneficial for protecting the host against ALV-J infection (Dai et al., 2016; Feng et al., 2016; Feng and Zhang, 2016; Li et al., 2017). To verify the findings of RNA-seq, we performed qRT-PCR to detect the expression levels of the key genes associated with MDA5-IFN*β*-JAK/STAT pathway. DOT1L-KO cells had increased mRNA levels of *IFIH1*, *IFNβ*, and *STAT1* and *MX1* upon ALV-J infection (Figure 6B), which was also observed through RNA-seq. Consistent with these findings, HD11 cells infected with ALV-J in the presence of 5 μM EPZ also revealed a significant upregulation of mRNA levels of *IFIH1*, *IFNβ*, and *STAT1* and *MX1* (Figure 6C). We, therefore, suggest that inhibition of DOT1L positively regulates *IFNβ* production *via* MDA5 pathways. Notably, MDA5 is an indispensable pattern recognition receptor (PRR) for RNA viruses, including ALV-J, to trigger the IFN response because RIG-I is absent in the chicken genome (Feng and Zhang, 2016; Li et al., 2017). Taken together, we propose that DOT1L-KO cells infected with ALV-J may facilitate enhanced viral dsRNA recognition and antiviral signaling activation mediated by MDA5.

### DOT1L Does Not Directly Regulate the MDA5-Mediated IFN*β* Signaling

**Figure 7:**
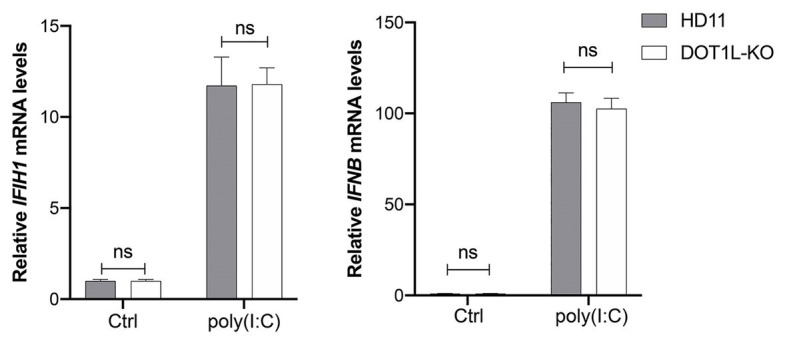
Disruptor of telomeric silencing 1-like does not directly regulate the melanoma differentiation-associated protein 5 (MDA5)-mediated IFNβ signaling. HD11 and DOT1L-KO cells were transfected with nothing (Ctrl) or 2 μM poly(I:C) for 8 h. The relative *IFIH1* and *IFNβ* mRNA levels were measured by qRT-PCR and normalized to GAPDH. All the data were shown as mean ± SD (error bars) from three independent experiments. ns, not significant; unpaired, two-tailed Student’s *t*-test.

To investigate whether DOT1L directly regulates the MDA5-mediated Type I interferon signaling, we transfected poly(I:C) into DOT1L-KO cells and WT HD11 cells to induce MDA5-mediated IFNβ transcription. Interestingly, the mRNA levels of MDA5 and IFNβ were not significantly changed when the DOT1L was knockout in HD11 cells. Meanwhile, the induction of MDA5-mediated IFNβ exhibited no statistically significant difference between DOT1L-KO and HD11 cells after the poly(I:C) stimulation (Figure 7). The results indicated that DOT1L might not simply targeting cellular components of MDA5-mediated IFNβ activation pathway. Therefore, we deduce that blocking DOT1L may disrupt the virus-host interaction that mediates immunosuppression and facilitates ALV-J infection.

### DOT1L Knockout Restores the Innate Immune Response to ALV-J Infection

**Figure 8:**
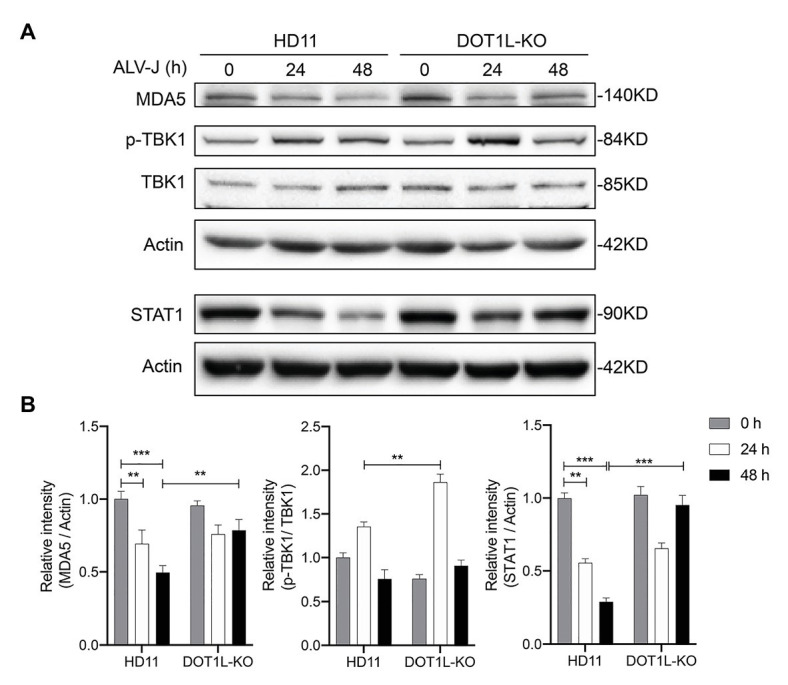
Disruptor of telomeric silencing 1-like deficiency restores the innate immune response to ALV-J infection in a time-dependent manner. **(A)** HD11 cells and DOT1L-KO cells were infected with ALV-J (MOI 2). Cell lysates were prepared at 0, 24, and 48 hpi for western blot to detect the protein expression levels of MDA5, total‐ and phospho-TBK1, STAT1, and β-actin. **(B)** Relative protein levels were quantified by densitometry using ImageJ software. All the data were shown as mean ± SD (error bars) from three independent experiments. ^**^p < 0.01; ^***^p < 0.001; unpaired, two-tailed Student’s *t*-test.

We next sought to assess the effect of DOT1L knockout on the protein levels of key proteins that are associated with the activation of innate antiviral signaling during viral infection. It has been reported that the expression of MDA5 was reduced in ALV-J infected chickens (Li et al., 2017); as expected, we also observed that the protein levels of MDA5 were markedly decreased in HD11 cells after ALV-J infection. Interestingly, our date show that DOT1L-knockout in HD11 cells relieved the viral mediated inhibition of the MDA5 protein (Figures 8A,B). Consistently, the phosphorylation of TBK1, which is involved in the MDA5-mediated IFNβ induction, was increased in DOT1L-KO HD11 cells compared to that in WT cells at 24 h post-infection with ALV-J (Figures 8A,B). In addition, we also found that ALV-J infection suppressed the expression of STAT1, a key upstream gene involved in antiviral innate immunity, which can be induced in a time-dependent manner after knocking out DOT1L in HD11 cells (Figures 8A,B). Taken together, these results demonstrate that DOT1L knockout restored the innate immune response toward ALV-J infection.

## Discussion

Avian leukosis viruses (ALVs) that infect chickens are currently classified into seven subgroups, including A, B, C, D, E, J, and K, based on the distinctive characteristics of the viral envelope glycoprotein. Because of its increased pathogenicity and transmission in comparison to other ALV subgroups, ALV-J is widespread in commercial meat-type chickens as well as egg-type chickens. The ALV-J genome contains two copies of a single-stranded RNA with positive polarity. Similar to other retroviruses, upon ALV-J infection, the single-stranded RNA viral genome is converted into a double-stranded DNA that subsequently integrates into the host genome (Lee et al., 2019). There is no doubt that ALV-J infection can alter the host chromatin state such as chromatin accessibility (Boehm and Ott, 2017), which probably facilitates its infection. However, little is known about the cellular chromatin regulators involved in ALV-J infection.

DOT1L is known as an epigenetic regulator that catalyzes the mono-, di-, and tri‐ methylation on lysine 79 of histone H3 (H3K79me1/2/3). Genome-wide ChIP-seq analysis indicated that DOT1L-mediated H3K79 methylation mainly distributes within the body of active genes (Godfrey et al., 2019). The role of DOT1L in chromatin regulation in mammalian cells has been well-elucidated (Nguyen and Zhang, 2011). Another well-studied function of DOT1L is its pro-tumorigenic effect, especially in the MLL-rearranged leukemia, and targeting DOT1L has therefore been proposed as a potential therapeutic approach (McLean et al., 2014; Boehm and Ott, 2017; Sarno et al., 2020). Recently, several reports have suggested that DOT1L and DOT1L-mediated H3K79 methylation are also been involved in the innate immunity and the inflammatory response (Chen et al., 2018; Kagoya et al., 2018; Marcos-Villar et al., 2018). However, the functional significance of DOT1L in ALV-J infection has not been reported.

In this study, we found that DOT1L expression as well as DOT1L-mediated H3K79me2 were significantly upregulated in chicken HD11 cells following ALV-J infection, which suggests that the expression of DOT1L is required for ALV-J replication. An important finding is that targeting DOT1L enzymatic activity with the small molecule inhibitor EPZ004777 results in obvious antiviral effects by decreasing ALV-J replication and virion production in ALV-J-infected HD11 cells (Figures 2C–E). Consistently, DOT1L deficiency made by CRISPR/Cas9 system-generated mutagenesis in HD11 cells also significantly reduced virus replication compared to that in WT cells (Figures 4A,B). Interestingly, recent studies by another group found that a DOT1L inhibitor or DOT1L knockdown can promote the viral propagation by decreasing the production of type-I interferons (IFNs), including IFN*β*, and inhibition of RIG-I-mitochondria-associated viral sensor (RIG-I-MAVS) association upon viral infection (Marcos-Villar et al., 2018, 2019, 2020). They also agree that DOT1L-mediated H3K79 methylation may have a very different mechanism by which it modulates the replication of influenza virus. It is known that ALV-J virus and influenza virus are very different although both belong to RNA viruses. ALV-J virus can integrate into the host genome but not into influenza virus (Marcos-Villar et al., 2018). Again, influenza virus is a strong inducer of Type I interferons, while the ALV-J virus used in our study is known as an immunosuppressive agent.

A previous study suggested that DOT1L silencing or inhibitor treatment suppresses IFN-β production triggered by TLR ligands and virus infection (Chen et al., 2018). However, in our study, blocking DOT1L did not significantly affect MDA5 and IFNβ induction after poly (I:C) stimulation (Figure 7), but greatly increased the cellular MDA5-IFNβ signaling after ALV-J infection (Figures 6A,B), suggesting that DOT1L inhibition may contribute to the recognition of ALV-J. In agreement with our findings, O’Connor et al. (2014) showed that DOT1L knockdown suppressed the replication of human cytomegalovirus (HCMV) but not that of adenovirus, suggesting that the effect of DOT1L in disrupting the life cycle of viruses may not be universally adopted. ALV-J is a poor inducer of innate immune responses, which are achieved by interactions with the host cell machinery, supporting the replication of the viral genome and the production of new virions. In our case, we deduced that blocking DOT1L may disrupt the virus-host interaction that facilitates ALV-J infection, but further studies are required.

In summary, our findings collectively show that targeted inhibition of DOT1L in chicken HD11 cells could restrict ALV-J infection by enhancing MDA5-IFNβ signaling, resulting in the activation of downstream ISGs and cytokine genes. Although further studies are still needed to elucidate the precise mechanisms of DOT1L action in ALV-J infection, our results clearly reveal that DOT1L is a novel candidate target for alleviating host immunosuppression caused by ALV-J infection. These findings may provide additional information about ALV-J replication in host cells and may lead to the development of DOT1L-based strategies for antiviral treatment in the future.

## Data Availability Statement

The datasets generated for this study have been deposited to the Sequence Read Archive (SRA) of NCBI. The BioProject accession number is PRJNA658491.

## Author Contributions

SC and HC conceived and designed the experiments and wrote and edited the manuscript. SC, DW, YL, RZ, and TW performed the experiments. SC, DW, XH, ZP, and HC analyzed the data. HC supervised the research project. All authors contributed to the article and approved the submitted version.

### Conflict of Interest

The authors declare that the research was conducted in the absence of any commercial or financial relationships that could be construed as a potential conflict of interest.

## Footnotes

^1^https://chopchop.cbu.uib.no/
